# Extracts from the Edible Mushroom *Sparassis crispa*: Nematicidal, Antimicrobial, and Antiviral Properties Supporting Its Functional Food Potential

**DOI:** 10.3390/foods15091559

**Published:** 2026-05-01

**Authors:** Marta Ziaja-Sołtys, Barbara Rajtar, Łukasz Świątek, Anna Biernasiuk, Katarzyna Dos Santos Szewczyk, Sebastian Granica, Andrzej Parzonko, Daniel Zalewski, Łucja Smolarska, Sebastian Skowron, Anna Bogucka-Kocka

**Affiliations:** 1 Chair and Department of Biology and Genetics, Medical University of Lublin, Chodźki 4a, 20-093 Lublin, Poland; daniel.zalewski@umlub.edu.pl (D.Z.);; 2 Department of Virology with Viral Diagnostics Laboratory, Medical University of Lublin, Chodźki 1, 20-093 Lublin, Polandlukasz.swiatek@umlub.edu.pl (Ł.Ś.);; 3 Chair and Department of Pharmaceutical Microbiology, Medical University of Lublin, Chodźki 1, 20-093 Lublin, Poland; 4 Department of Pharmaceutical Botany, Chair of Pharmacognosy and Pharmaceutical Botany, Medical University of Lublin, Chodźki 1, 20-093 Lublin, Poland; 5 Department of Pharmaceutical Biology, Faculty of Pharmacy, Medical University of Warsaw, 1 Banacha Street, 02-097 Warsaw, Polandandrzej.parzonko@wum.edu.pl (A.P.)

**Keywords:** *Sparassis crispa*, medicinal mushroom, nematicidal activity, antiviral activity, antibacterial activity, bioactive metabolites

## Abstract

*Sparassis crispa* (cauliflower mushroom) is an edible medicinal fungus known for its diverse array of bioactive metabolites. Despite its established nutritional and pharmacological relevance, its antimicrobial, antiviral, and antiparasitic activities remain insufficiently investigated. In the present study, extracts of the fruiting bodies of *S. crispa* were prepared using four solvents (water, 60% ethanol, methanol–acetone–water [3:1:1], and 1% acetic acid) and evaluated for their chemical composition and broad-spectrum biological activities. UHPLC-MS/MS profiling revealed distinct metabolite profiles among the extracts, including identification of nucleosides such as adenosine and methylthioadenosine. All extracts exhibited nematicidal activity against *Rhabditis* sp. nematodes in a dose-dependent manner, with the 60% ethanol extract being the most potent (LD_50_ = 4.2 mg/mL). In antiviral assays, the water extract partially inhibited Coxsackievirus B3 (CVB3) replication, reducing infectious titers by approximately 2 log units, whereas none of the extracts showed a significant effect against *Herpes simplex* virus type 1 (HSV-1). Antibacterial testing demonstrated activity only for the 1% acetic acid extract, which inhibited several Gram-positive and Gram-negative bacteria at minimum inhibitory concentrations of 10–20 mg/mL. No antifungal activity against *Candida* spp. was observed. These findings identify *Sparassis crispa* as a promising edible source of bioactive compounds, exhibiting pronounced nematicidal and moderate antimicrobial activities, and support its potential application in the development of functional foods and nutraceuticals. They further justify targeted isolation and mechanistic studies to characterize the metabolites responsible for these effects and to clarify their relevance for food-based health promotion.

## 1. Introduction

*Sparassis crispa* (Wulf.) (Fr.), commonly known as the cauliflower mushroom (*Hanabiratake* in Japan), is an edible basidiomycete fungus in the family *Sparassidaceae*. It is widely distributed in temperate forests of Europe, Asia, and North America, where it grows as a wood-decaying parasite on coniferous trees, including *Larix kaempferi*, *Pinus koraiensis*, *Pinus nigra*, and *Pinus sylvestris* [1,2]. Morphological characterization combined with rDNA sequence analysis has demonstrated clear differences between Asian and European/North American populations of *S. crispa* [3,4]. Molecular studies examining correlations between morphology and geographic distribution further support the division of the genus into three groups: *S. crispa* from Europe and eastern North America, *S. radicata* from western North America, and *S. latifolia* from Asia [5,6,7]. Among these, *S. crispa* remains one of the most extensively studied taxa due to its nutritional value and medicinal potential [8,9,10].

**Figure 1:**
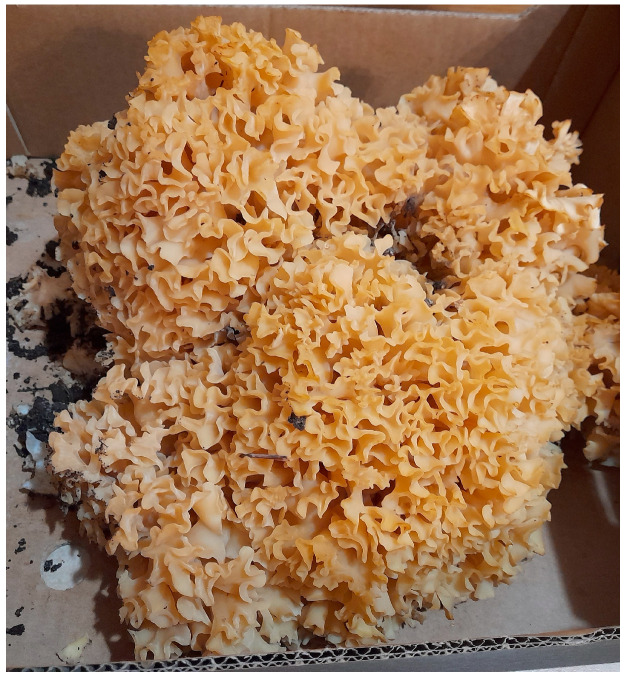
*Sparassis crispa* fruiting body. Own archive.

The fruiting bodies of *S. crispa* exhibit a characteristic cauliflower-like morphology, creamy-white coloration (Figure 1), and mild aromatic flavor associated with volatile metabolites such as 3-octanone, DL-3-octanol, and 1-octen-3-ol [3,11,12,13]. Ecologically, the species functions as a weak parasite and saprotroph contributing to lignocellulosic decomposition and thereby influencing forest ecosystem processes [14].

Beyond its ecological role, *S. crispa* is garnering scientific interest due to its notable nutritional value and medicinal potential. This species is characterized by an exceptionally high polysaccharide content, with β-glucans comprising approximately 30–40% of its dry weight, a feature considered to contribute substantially to its reported health-promoting effects [3,15,16]. Indeed, *S. crispa* exhibits a wide range of reported biological activities, including anti-tumor, immunomodulatory, hematopoietic, anti-inflammatory, antioxidant, anti-diabetic, and wound-healing effects [3]. Most studies to date have focused on its β-glucans as key bioactive constituents conferring anticancer and immune-enhancing properties [3]. These β-glucans, together with other heteropolysaccharides containing fucose, galactose, glucose, mannose, and xylose, have been shown to act as biological response modifiers with antiproliferative and immunostimulatory effects, evidenced by in vitro and in vivo models demonstrating anticancer activity (e.g., against colon cancer cells) and neuroprotective effects in glutamate toxicity models [17,18,19,20].

In addition to polysaccharides, *S. crispa* produces an array of low-molecular-weight secondary metabolites, such as sesquiterpenoids, maleic acid derivatives, and benzoic acid derivatives, which further contribute to its pharmacological profile. Notably, crystalline antibiotic compounds, including methyl 2-hydroxy-4-methoxy-6-methylbenzoate, known as sparassol, and related benzoates, have been isolated from *S. crispa* cultures [3,4]. Sparassol is credited with antimicrobial activity and has also been implicated in antiparasitic effects. An ethyl acetate fraction rich in sparassol from a *Sparassis* species showed potent nematicidal activity against the pine wood nematode (*Bursaphelenchus xylophilus*), with LC_50_ values in the low μg/mL range [21]. This diverse repertoire of bioactive metabolites underscores *S. crispa* therapeutic relevance.

Furthermore, *S. crispa* has demonstrated antimicrobial potential, with earlier studies reporting that its extracts or pure compounds can inhibit various bacteria and fungi; for example, sparassol isolated from *S. crispa* showed antifungal activity against *Candida albicans* and antibiotic effects against certain bacteria [22,23]. Nonetheless, several aspects of *S. crispa* bioactivity remain insufficiently studied. There is a knowledge gap regarding its efficacy as an antiparasitic (especially anthelmintic) agent, as well as its antiviral spectrum and broad antibacterial effects. Given the emergence of drug-resistant infections and the need for new therapeutics, examining such activities in *S. crispa* is of considerable interest.

The aim of this study was to evaluate the broad spectrum of biological activities of *S. crispa*, with particular emphasis on their antiparasitic, antimicrobial, and antiviral properties. We prepared extracts from *S. crispa* fruiting bodies using solvents of different polarity and initially characterized the chemical composition of two selected extracts by UHPLC–MS/MS profiling to identify metabolites potentially associated with the observed bioactivities. We then assessed each extract for nematicidal activity against free-living nematodes of the genus *Rhabditis*, antiviral activity against CVB3 (a human enterovirus) and HSV-1, and antimicrobial activity against a panel of pathogenic bacteria and *Candida* yeasts.

The selection of viral, bacterial, and nematode models in this study was based on their biological relevance and suitability for evaluating the broad-spectrum activity of the *Sparassis crispa* extracts. Coxsackievirus B3 (CVB3) and *Herpes simplex* virus type 1 (HSV-1) were included as representative RNA and DNA viruses, respectively, enabling assessment of antiviral effects across distinct replication mechanisms and structural types relevant to human infections. The bacterial panel included both Gram-positive and Gram-negative species, enabling assessment of antimicrobial activity against organisms with distinct cell wall structures and permeability barriers, which are critical determinants of susceptibility. The use of standardized reference strains further supports the comparability of our results with previously published data. Nematodes of the genus *Rhabditis* have been reported as opportunistic agents capable of infecting humans, with documented pathogenic involvement in the external auditory canal, gastrointestinal tract, urinary system and, in rare cases, the central nervous system [24,25,26,27]. Owing to their ecological prevalence, *Rhabditis* spp. provide a convenient experimental model. Their physiological characteristics make them a suitable proxy for parasitic nematodes of both medical and agricultural relevance, particularly in the context of pre-liminary bioactivity screening.

Together, these models form a complementary system for assessing antiviral, antibacterial, and nematicidal properties, supporting a comprehensive evaluation of the functional potential of *Sparassis crispa*. The results are intended to support the evaluation of *S. crispa* as a source of bioactive compounds relevant to functional food and nutraceutical applications, while also providing a basis for future studies focused on the isolation, identification, and mechanistic evaluation of active ingredients.

## 2. Materials and Methods

### 2.1. Fungal Material

The fruiting bodies of the fungus *S. crispa* were collected in forests near Opole Lubelskie, Poland, in August 2022. Immediately after collection, the fruiting bodies were cleaned and dried in a mushroom dryer (Optimum KN-128E, Sencor, Ricany, Czech Republic). The drying process took 2–3 days. Then, the dried raw material was ground in an electric grinder IKA-WERKE M20 (IKA-Werke GmbH, Staufen, Germany) and divided into tightly closed foil bags. The material prepared in this way was stored in the freezer at −20 °C.

The taxonomic identification of the collected fungal material was performed based on macroscopic morphological characteristics, including the typical cauliflower-like, highly branched basidiocarp structure, coloration, and growth form, which are diagnostic features of *Sparassis crispa*. Species determination was carried out by an experienced mycologist prof. Anna Bogucka-Kocka familiar with macrofungi of the genus *Sparassis*, using standard taxonomic keys and reference descriptions available in the mycological literature. The identification was further verified by comparison with documented morphological traits reported for *S. crispa* in Europe. Although no voucher specimen was deposited and molecular confirmation was not performed, the identification was considered reliable based on expert taxonomic assessment and field experience.

### 2.2. Extracts Preparation

For extraction, 10 g of dried *S. crispa* was used. Extracts were prepared using a mixture of methanol–acetone–water (3:1:1, *v*/*v*/*v*; 3 × 80 mL; MAW), 60% ethanol (3 × 80 mL; ET60%), 1% acetic acid (3 × 80 mL; 1% AA), and water (3 × 80 mL; W). During extraction with methanol–acetone–water (3:1:1) and 1% acetic acid, samples of *S. crispa* were sonicated at a controlled temperature (45 ± 2 °C) for 30 min. Ethanolic and aqueous extractions were performed by shaking at room temperature for 24 h. The combined extracts were filtered through Whatman filter paper, concentrated under reduced pressure, and after freezing, lyophilized in a vacuum concentrator (Free Zone 1 apparatus; Labconco, Kansas City, KS, USA) to obtain dried residues.

### 2.3. UHPLC-MS/MS Analysis

Ultra-high-performance liquid chromatography analysis was conducted using the Ultimate 3000 series system (Dionex, Idstein, Germany), coupled with an Amazon SL ion trap mass spectrometer (Bruker Daltonik GmbH, Bremen, Germany). Chromatographic separation was achieved on a Kinetex XB-C18 analytical column (150 mm × 2.1 mm, 1.9 µm particle size; Phenomenex, Torrance, CA, USA). The column temperature was maintained at 25 °C throughout the analysis. The mobile phase consisted of solvent A (0.1% formic acid in deionized water) and solvent B (0.1% formic acid in LC-MS grade acetonitrile). The mobile phase was delivered at a flow rate of 0.300 mL/min. A gradient elution program was employed as follows: 0 min–1% B, ramping to 26% B at 60 min. The column was equilibrated for 7 min between successive injections. An injection volume of 5 µL was utilized for each sample. Mass spectrometric detection was performed using an electrospray ionization (ESI) interface operating in both positive and negative ionization modes. The ESI source parameters were set as follows: nebulizer pressure, 45 psi; drying gas flow rate, 9 L/min; drying temperature, 134 °C; and capillary voltage, 4.5 kV. Data acquisition was conducted over a mass-to-charge (*m*/*z*) range of 70–2200. Analytes were detected by monitoring their pseudomolecular ions. Tentative compound identification was performed by comparing the obtained mass spectra with previously reported data in the scientific literature.

### 2.4. Determination of Antinematicidal Activity

The research model was a *Rhabditis* sp. nematode culture from the collection of the Department of Biology and Genetics, Medical University of Lublin. In vitro nematode cultures were grown in sterile 6-well plates on solid agar medium (Difco™ Granulated Agar, Becton Dickinson, NJ, USA) supplemented with fetal bovine serum (FBS, EURyx) and incubated for 5 days at room temperature (21 °C) to obtain all developmental stages. The nematodes were then eluted from the agar medium with 0.6% NaCl solution. The nematode suspension was transferred to sterile 24-well plates at 400 µL per well. Stock solutions of the tested extracts were prepared at concentrations of 200 and 100 mg/mL. Aliquots of extracts (50 μL) were added to the cultures, yielding final concentrations ranging from 1.4 to 22.2 mg/mL (1.4, 2.7, 5.5, 11.1, and 22.2 mg/mL). Dilutions of the tested fractions were prepared in distilled water. The commercially available antiparasitic drug mebendazole was added to the positive control culture (final concentrations: 1.4–22.2 mg/mL), while only distilled water (50 μL) was added to the negative control. Mebendazole concentrations were experimentally selected to correspond to the range used for the tested fractions. After 24 h of exposure at room temperature (21 °C), the nematodes were examined under optical and stereoscopic microscopy. Viability, morphological deformations, and developmental abnormalities were assessed. Larval and adult survival was assessed based on behavioral parameters, including motility, reproductive activity, and the adult-to-larval ratio. Live and dead worms in liquid cultures were counted on microscope slides. The number of live and dead nematodes was recorded for each preparation, and their sum equaled 100%. All control samples were processed identically to the experimental preparations [28]. The experiment was performed in three independent replicates.

The obtained results were statistically analyzed using a one-way ANOVA (GraphPad 10.4.1 (627) Software, San Diego, CA, USA). Results with *p* value < 0.05 were considered statistically significant.

### 2.5. Determination of Antiviral Activity

#### 2.5.1. Cell Culture and Viruses

The study used the Vero cell line (American Type Culture Collection, ATCC; CCL-81) derived from normal kidney cells of an adult African green monkey. Dulbecco’s Modified Eagle’s Medium (DMEM) (Corning, New York, NY, USA) with the addition of 10% fetal bovine serum (FBS, Corning, New York, NY, USA) and antibiotics (Penicillin–Streptomycin Solution 100×; Corning, New York, NY, USA) was used for cell culture. The Vero cell culture was incubated at 37 °C in a 5% CO_2_ atmosphere (CO_2_ incubator, Panasonic Healthcare Co., Tokyo, Japan). To test the antiviral activity of the extracts, Coxsackievirus B3 (CVB3; ATCC VR-30) and *Herpes simplex* virus type 1 (HSV-1; ATCC VR-260) were used. The viruses were multiplied in the Vero cell line at 37 °C, and then their suspensions were stored at −80 °C until further tests were carried out. The viral infectious titer was determined using an endpoint dilution assay. For virus titration, the Vero cells were cultured for 24 h at 37 °C in 96-well flat-bottom plates. Afterward, culture medium was removed from 96-well plates, and tenfold dilutions (6 replicates) of each titrated virus were incubated with Vero cells for 72 h. Daily observations were conducted to monitor the progression of cytopathic effect (CPE). After incubation, all media were discarded, and the infectious titer CCID_50_ (50% cell culture infectious dose; a dose capable of inducing CPE in 50% of infected cells) of each virus was quantified by measuring the reduction in cellular viability of infected cells using the MTT method described in Section 2.5.3.

#### 2.5.2. Preparation of *Sparassis crispa* Mushroom Extracts for Antiviral Testing

The dried mushroom extracts were dissolved in deionized water to a final concentration of 200 mg/mL. The dissolved extracts were sterilized using SARSTEDT disposable filters with a pore diameter of 0.2 μm and stored at minus 20 °C until testing.

#### 2.5.3. Cytotoxicity Assay

The cell suspension was seeded (100 µL/well) into 96-well plastic plates (Becton Dickinson and Company, Franklin Lakes, NJ, USA) at a density of 1.5 × 10^4^ cells per well. After 24 h of incubation at 37 °C, the medium was removed, and the cells were treated with the examined extracts diluted in culture medium containing 2% serum. Two-fold serial dilutions of compounds, from 40 to 0.0195 mg/mL, were added to the cells in triplicate. The cell cultures were incubated for 72 h at 37 °C in a 5% CO_2_ atmosphere.

After incubation with the tested extracts, the culture medium was removed, and the cells were washed with PBS (phosphate-buffered saline, Corning, New York, NY, USA). Subsequently, 100 μL of culture medium containing 10% MTT (Methyl Thiazolyl Tetrazolium) solution (5 mg/mL in PBS; Sigma-Aldrich, Saint Louis, MO, USA) was added to each well, and the plates were incubated for an additional 4 h at 37 °C. Then, 100 μL of solvent containing 50% dimethylformamide (POCH, Gliwice, Poland) and 20% SDS (sodium dodecyl sulfate, 99% pure, AppliChem, Darmstadt, Germany) in PBS was added to dissolve the MTT formazan precipitates. After overnight incubation and formazan dissolution, absorbance was measured in a 96-well plate reader at 540 and 620 nm (Synergy H1 Multi-Mode Microplate Reader, BioTek, Winooski, VT, USA). Based on the obtained measurements, the CC_50_ and CC_10_ cytotoxic concentrations, defined as the concentration of the tested extracts that caused a 50% or 10% decrease in cell activity relative to the untreated control, were determined using Gen 5 software (ver. 3.09.07; BioTek, Winooski, VT, USA) [29]. Cytotoxicity of the extracts was assessed to identify non-toxic doses for testing their antiviral activity.

#### 2.5.4. Antiviral Assay

The infectious titer of HSV-1 used in this study was 5.5 ± 0.25 log CCID50/mL, and the CVB3 titer was 5.85 ± 0.28 log CCID50/mL, respectively. After removing the medium from a 24 h culture of Vero cells in 96-well plates, a suspension of the tested virus (HSV-1 or CVB3) was added at a 100-fold CCID50/mL dose. After 1 h of incubation at 37 °C to allow virus attachment and penetration, the infected media were removed from the culture, and the remaining cells were washed with PBS. Then, the tested extracts and appropriate reference antiviral substances were added to the infected cell culture; acyclovir (ACV) (Sigma-Aldrich, Saint Louis, MO, USA) was used for HSV-1, and ribavirin (RBV) (Sigma-Aldrich, Saint Louis, MO, USA) for CVB3. Extracts from the stock solution were diluted in medium containing 2% serum to non-toxic concentrations for cells. Noncytotoxic concentrations were the highest concentrations of the examined extract at which cell viability after 72 h of incubation was at least 90%. The virus control consisted of cultures to which only medium without extracts was added. After 72 h of incubation at 37 °C in the presence of 5% CO_2_, the appearance of 100% cytopathic effect (CPE) in the virus control was assessed using an inverted light microscope (CKX41; Olympus Corporation, Tokyo, Japan), equipped with a camera (Moticam 3+, Motic, Hong Kong) and image documentation software (Motic Images Plus 2.0, Motic).

Infected cells were frozen three times at −70 °C, thawed, and then the viruses were titrated. Viral titration was performed using the endpoint dilution assay, as described in Section 2.5.2. For virus titration, the Vero cells were cultured for 24 h at 37 °C in 96-well flat-bottom plates. Briefly, the culture medium was removed, and 10-fold dilutions of the virus samples in the presence of the tested extracts or reference compounds, or in the absence of the tested substances (virus control), were added. The plates were incubated for 72 h at 37 °C until a typical CPE was visible using an inverted microscope. Viral titers (CCID_50_) were calculated using the MTT method. Finally, the difference between the control virus titer (CVB3 or HSV-1) expressed in log_10_ and the virus titer treated with the tested extract or reference substance (ribavirin or acyclovir) was calculated. The measure of antiviral activity was the reduction in virus titer (Δlog) by the extract or compounds compared with the virus control (Δlog = logCCID_50_VC − logCCID_50_TS; VC—virus control, TS—tested sample (extract or reference antiviral)) [30].

The viral loads of CVB3 and HSV-1 were measured in samples collected from antiviral assays using qPCR (quantitative PCR) and RT-qPCR (reverse transcriptase quantitative PCR), respectively. The RNA of CVB3 and DNA of HSV-1 was isolated using QIAamp Viral RNA Mini Kit (Cat.: 52904 QIAGEN GmbH, Hilden, Germany) and QIAamp DNA Mini Kit (Cat.: 51304 QIAGEN GmbH, Hilden, Germany), respectively. The RNA isolates of CVB3-infected cells were subjected to one-step RT-qPCR amplification with iTaq Universal SYBR Green One-Step Kit (Cat.: 1725150, Bio-Rad Laboratories, Life Science Group, Hercules, CA, USA) and enterovirus-specific primers entrinR (5′-GAAACACGGACACCCAAAGTA-3′) and entrinF (5′-CGGCCCCTGAATGCGGCTAA-3′) on the CFX96 thermal cycler (Bio-Rad Laboratories). The parameters of RT-qPCR amplification were as follows: reverse transcription (50 °C, 10 min), activation of polymerase (95 °C, 1 min), cycling (40 repeats: denaturation (95 °C, 10 s), annealing and synthesis (65 °C, 30 s), fluorescence acquisition), and melting curve analysis (65–95 °C, 0.5 °C increment/5 s).

The DNA isolates from anti-HSV-1 assays were subjected to qPCR amplification with SsoAdvanced Universal SYBR Green Supermix (Bio-Rad Laboratories) and primers (UL54F–5′ CGCCAAGAAAATTTCATCGAG 3′, and UL54R–5′ ACATCTTGCACCACGCCAG 3′) on the CFX96 thermal cycler. The qPCR parameters were as follows: polymerase activation (98 °C, 3 min); cycling (40 repeats: DNA denaturation (95 °C, 10 s); annealing and synthesis (60 °C, 30 s), fluorescence acquisition); melting curve analysis (65–95 °C). The CVB3 and HSV-1 viral loads in the extract-treated samples were calculated by comparing them with VC (virus control) using the relative quantity (ΔCq) method on CFX Manager™ Dx Software version 3.1.3090.1022 (Bio-Rad Laboratories) [31].

The experiments were performed three times. Means and standard deviations were calculated from the numerical data. All assays were analyzed using one-way Analysis of Variance (ANOVA) with Tukey’s post hoc test (*p* < 0.05) in Statistica 9.1.

### 2.6. Antimicrobial Assay

The extracts prepared from *S. crispa* were tested in vitro for antimicrobial effects using the broth microdilution method according to the European Committee on Antimicrobial Susceptibility Testing (EUCAST) [32] and Clinical and Laboratory Standards Institute guidelines. The activity towards twenty reference strains of microorganisms was studied, including Gram-positive bacteria, Gram-negative bacteria, and fungi belonging to yeasts. All strains used in this study were obtained from the American Type Culture Collection (ATCC) or the Centers for Disease Control (CDC) (Table 1).

First, the microbial cultures were grown on nutrient agar (BioMaxima, Lublin, Poland) or Sabouraud dextrose agar (BioMaxima, Lublin, Poland) at 35 °C for 18–24 h or 30 °C for 24–48 h for bacteria and fungi, respectively. Tested extracts were dissolved in water (100 mg/mL). The inhibition of bacterial and fungal growth was judged by comparison with a control culture without any studied sample. Ciprofloxacin, vancomycin and nystatin (Sigma, St. Louis, MA, USA) were used as a reference antimicrobial drugs.

The MIC (Minimum Inhibitory Concentration) value of these extracts was investigated by their two-fold microdilution broth method in Mueller–Hinton broth (bioMaxima, Lublin, Poland) (for bacteria) and RPMI 1640 broth with MOPS (morpholinepropanesulfonic acid) (Sigma, St. Louis, MA, USA) (for fungi). The study was conducted in 96-well polystyrene plates. The final concentrations of the tested extracts ranged from 0.15 to 20 mg/mL. Suspensions of bacteria and fungi were prepared in 0.85% NaCl with an optical density of 0.5 McFarland standard. Next, individual suspensions of microorganisms were added to each well containing broth and different concentrations of the extracts. After incubation, the MIC was analyzed spectrophotometric as the lowest concentration of the extract with complete bacterial or fungal growth inhibition. Appropriate water, growth, and sterility controls were prepared. The medium without extracts from *S. crispa* was used as control.

The MBC (Minimum Bactericidal Concentration) or MFC (Minimum Fungicidal Concentration) were defined as the lowest concentration of the studied extract that is necessary to kill the bacteria or fungus, respectively. MBC and MFC were determined by taking cultures from each well after determining the MIC and plating them on an appropriate agar medium. The plates were then incubated under the appropriate conditions. The lowest extract concentrations without visible growth were assessed as bactericidal or fungicidal. The experiments were repeated three times, and representative data are presented as mean and standard deviation (+/−SD). Additionally, MBC/MIC and MFC/MIC values were calculated in order to determine the bactericidal/fungicidal (MBC/MIC ≤ 4, MFC/MIC ≤ 4) or bacteriostatic/fungistatic (MBC/MIC > 4, MFC/MIC > 4) effects of these extracts [33,34].

## 3. Results

### 3.1. Extraction Efficiency

The efficiency of the extraction process of 10 g of dry mass of the *S. crispa* mushroom using four different solvents, i.e., 60% ethanol (ET60%), methanol–acetone–water mixture (3:1:1, *v*/*v*/*v*, MAW), 1% acetic acid (1% AA), and water (W), was similar. The extracts were concentrated in a vacuum evaporator under reduced pressure. The extraction efficiencies obtained for the individual solvents are presented in Table 2.

### 3.2. Results of UHPLC Analysis

The UHPLC analysis was performed for water (W) and 60% ethanolic (ET60%) extracts from *Sparassis crispa*. The analysis revealed the presence of 17 chromatographic features and demonstrated noticeable variability in the qualitative and relative composition of the studied extracts (Figure S1), which can be attributed to differences in polarity and solubility of metabolites in the applied extraction solvents. The detailed data obtained from diode array detection (DAD) and mass spectrometry (MS) are presented in Table 3.

The UHPLC–ESI–MS analysis further indicated a complex but only partially characterized metabolite profile. Due to the non-targeted nature of the approach and the lack of authentic reference standards for most detected features, the majority of signals could only be assigned at the level of putative annotation or compound class according to the Metabolomics Standards Initiative (MSI Level 2–3). Therefore, the present study should be considered as a preliminary metabolic fingerprinting rather than a full structural elucidation of the extract composition [35].

Based on a comparison of the literature and characteristic pseudomolecular ions, a limited number of compounds were tentatively identified. Among them, a signal at *m*/*z* 268 in positive ion mode was assigned to adenosine, while a feature at *m*/*z* 298 was attributed to 5′-methylthioadenosine. These nucleoside derivatives are frequently reported in basidiomycetes and are consistent with the conserved purine metabolism in fungi. In addition, selected signals corresponding to low-molecular-weight phenolic acids, including vanillic- and syringic acid-related derivatives, were observed, which is consistent with previously reported aromatic metabolites in edible mushroom species.

Several higher-mass features (e.g., *m*/*z* 369, 488, 531, and 611) were detected in both extracts and were classified as conjugated phenolic-like compounds or complex secondary metabolites. However, their exact structural elucidation was not possible under the experimental conditions used. Consequently, these signals are currently interpreted as metabolite classes rather than individual, defined structures.

Importantly, signals that initially resembled plant-derived flavonoids were critically re-evaluated. Considering that basidiomycetes are not generally known to produce classical flavonoid structures, these features are more plausibly attributed to structurally related phenolic derivatives or fungal-specific conjugated metabolites. Nevertheless, this assignment remains tentative and requires further confirmation using targeted MS/MS experiments and authentic reference standards.

Overall, the metabolite profile observed in *S. crispa* extracts reflects a chemically diverse system dominated by nucleoside derivatives and phenolic-type metabolites. However, the present UHPLC–ESI–MS data should be interpreted as a preliminary chemical fingerprint, providing a foundation for future targeted isolation and structural characterization of these compounds.

**Table 3:** Putative identification of metabolites detected in *Sparassis crispa* using LC–ESI–MS analysis.

No.	Rt (min)	*m*/*z* (Ion)	Proposed Formula	Putative Identification	Compound Class	MSI Level	Ref.
1	1.4	339 [M+H]^+^	~C13H18N4O7	Modified nucleoside derivative	Nucleosides	Level 3	[36]
2	1.9	132 [M+H]^+^	~C5H10NO3	Amino acid (unresolved isomer)	Primary metabolite	Level 3	[37]
3	2.3	262 [M+H]^+^	C10H12N4O5	Inosine	Nucleosides	Level 2	[36]
4	2.5	291 [M−H]^−^	C15H14O6	Phenolic compound (unresolved; non-flavonoid)	Phenolics	Level 3	[37]
5	2.9	294 [M+H]^+^	~C14H14O7	Phenolic acid derivative	Phenolics	Level 3	[37]
6	3.7	611 [M−H]^−^	~C27H30O16	High-mass conjugated phenolic (tentative)	Polyphenols	Level 3	[3]
7	4.0	268 [M+H]^+^	C10H13N5O4	Adenosine	Nucleosides	Level 1	[8]
8	4.4	369 [M−H]^−^	~C16H18O10	Caffeoyl-related conjugate	Phenolics	Level 3	[38]
9	5.1	166 [M+H]^+^	C8H6O4	Vanillic acid/isomer	Phenolic acids	Level 2	[38]
10	6.0	284 [M+H]^+^	C15H10O6	Phenolic metabolite (non-flavonoid)	Phenolics	Level 3	[38]
11	6.7	328 [M+H]^+^	~C18H18O6	Putative secondary metabolite	Unknown (phenolic/terpenoid)	Level 3	[38]
12	8.1	531 [M−H]^−^	~C26H28O12	Conjugated metabolite (tentative)	Polyphenols	Level 3	[3]
13	9.0	488 [M−H]^−^	~C23H20O12	Conjugated phenolic derivative	Polyphenols	Level 3	[38]
14	11.5	205 [M+H]^+^	~C11H12O4	Sparassol or derivative	Phenolic metabolites	Level 2	[4]
15	14.7	298 [M+H]^+^	C11H15N5O3S	5′-Methylthioadenosine	Nucleosides	Level 1	[8]
16	15.2	384 [M+H]^+^	C22H26NO5	Veracintine	Alkaloids	Level 2	[36]
17	33.0	197 [M−H]^−^	C9H10O5	Syringic acid/isomer	Phenolic acids	Level 2	[39]

Retention time (Rt), UV–Vis absorption maxima, and mass spectrometric data (positive and negative ion modes) were used for compound annotation. Proposed molecular formulas were assigned based on nominal mass values due to the absence of high-resolution measurements. Metabolite identification levels were defined according to the Metabolomics Standards Initiative (MSI): Level 1—identified compounds confirmed with reference standards; Level 2—putatively annotated compounds based on spectral similarity and literature data; Level 3—tentatively characterized compound classes. Assignments were supported by comparison with published data and known metabolite profiles of *S. crispa* [35].

### 3.3. Nematicidal Activity

The present study evaluated the nematicidal properties of four extracts derived from the fruiting bodies of *S. crispa*, namely aqueous (water, W), 60% ethanol (ET60%), methanol–acetone–water (MAW), and 1% acetic acid extracts (1% AA), (Patent, Poland no.232918). To the authors’ knowledge, this is the first report assessing the antiparasitic potential of these specific extract types obtained from *S. crispa* fruiting bodies. The assays were conducted using *Rhabditis* sp. as a model organism, with nematodes exposed to extract concentrations of 1.4, 2.7, 5.5, 11.1, and 22.2 mg/mL for 24 h. Viability in the control group cultured in distilled water reached 98%, confirming suitable baseline conditions for biological comparison. LD_50_ is the amount of ingested substance that kills 50% of a tested nematode sample. LD_50_ values were read from the graphs based on the mean % viability values obtained from three replicates.

LD_50_ values were estimated on the basis of dose–response relationships derived from mean nematode viability data and obtained by graphical interpolation from the fitted curves presented in the figures. This approach is commonly applied in preliminary in vitro screening studies involving nematicidal activity, particularly when using discrete concentration points and biological material exhibiting inherent variability. It should be noted that the resulting LD_50_ values represent approximate indicators of biological activity rather than exact pharmacological parameters. More advanced nonlinear regression models may provide higher statistical precision; however, the present study was designed as an exploratory assessment of nematicidal potential, and the applied method was considered sufficient for comparative evaluation of the tested samples.

**Figure 2:**
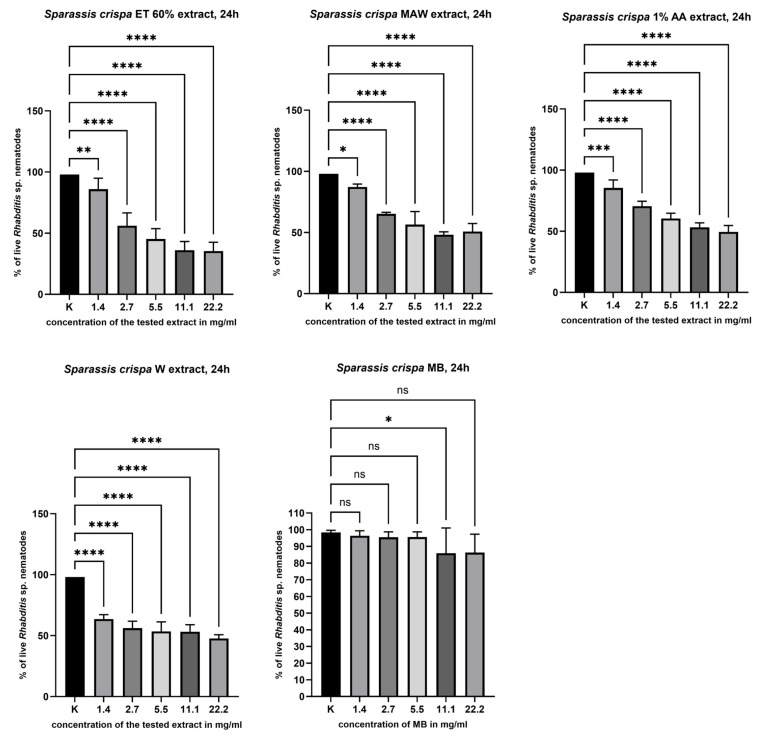
Survival of *Rhabditis* sp. nematodes (%) after 24 h incubation against aqueous (W), aqueous–acetic (1% AA), ethanol (ET60%) and methanol–acetone–aqueous (MAW) extracts of *S. crispa* fruiting bodies and against the drug mebendazole (MB) as a positive control. ****—*p* < 0.0001, ***—0.0001 < *p* < 0.001, **—0.001 < *p* < 0.01, *—0.01 < *p* < 0.05, ns—not significant (*p* ≥ 0.05). Error bars mark standard deviation.

Based on the experimental findings, the viability of *Rhabditis* sp. nematodes markedly declined following 24 hours of exposure to the water extract (W) derived from the fruiting bodies of *S. crispa* (Figure 2). The LD_50_ value for the extract was calculated at 15 mg/mL. The strongest nematicidal effect was observed at a concentration of 22.2 mg/mL, which reduced nematode viability to 47.71%. Lower concentrations of 11.1 mg/mL and 5.5 mg/mL resulted in viability levels of 53.18% and 53.5%, respectively. Statistical analysis confirmed the significance of these differences. Notably, the extract also exhibited appreciable activity at concentrations of 2.7 mg/mL and 1.4 mg/mL, where nematode viability remained at 56.14% and 63.62%, respectively.

**Figure 3:**
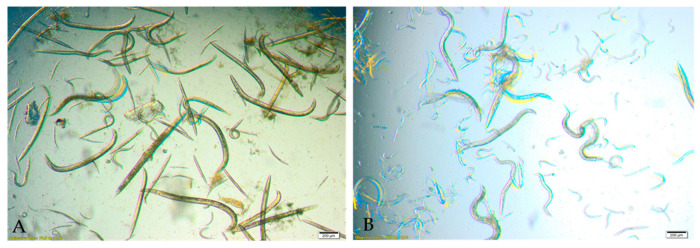
Nematodes of the genus *Rhabditis* sp. after 24 h exposure to a 60% ethanol extract of the fruiting bodies of the fungus *S. crispa* at the tested concentrations of 22.2 mg/mL (**A**); the control sample (**B**). Photos from plate wells, taken under a CXK 41 inverted optical microscope (Olympus) at 40× magnification. Scale bar length equal to 200 μm.

Exposure of *Rhabditis* sp. nematodes to the 60% ethanol extract for 24 h resulted in a pronounced and statistically significant reduction in viability (Figure 2). The LD_50_ value determined after 24 h was 4.2 mg/mL. The strongest nematicidal activity was recorded at concentrations of 22.2 mg/mL and 11.1 mg/mL, where the proportion of viable nematodes decreased to 35.26% and 36.03%, respectively. Slightly weaker effects were observed at concentrations of 5.5 mg/mL, yielding 45.18% viability, as well as at 2.7 mg/mL and 1.4 mg/mL, where viability reached 56.0% and 85.97%, respectively (Figure 2). Additionally, although numerous nematodes remained alive across these treatments, many exhibited impaired motility, characterized by reduced movement or partial paralysis, particularly in the posterior region of the body (Figure 3).

**Figure 4:**
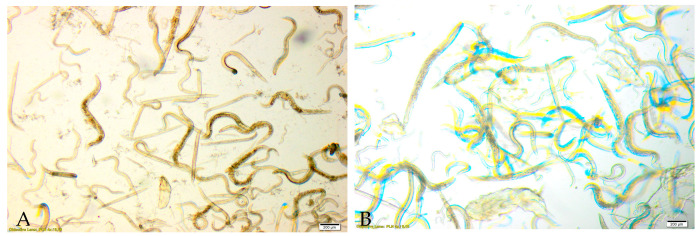
Nematodes of the genus *Rhabditis* sp. after 24 h exposure to methanol-acetone-water (MAW) extract from fruiting bodies of the fungus *S. crispa* at the tested concentrations of 22.2 mg/mL (**A**); the control sample (**B**). Photos of preparations taken under a BX53 optical microscope (Olympus) at 40× magnification. Scale bar length equal to 200 μm.

Nematodes of the genus *Rhabditis* sp. also exhibited sensitivity to the methanol–ethanol–water (MAW) extract (Figure 4). The LD_50_ value determined after 24 h of exposure was 9.6 mg/mL. At the highest tested concentration (22.2 mg/mL), nematode viability was reduced to 50.73%. At concentrations of 11.1 mg/mL, 5.5 mg/mL, 2.7 mg/mL, and 1.4 mg/mL, viability values were 48.21%, 56.46%, 65.22%, and 87.21%, respectively. All observed effects were confirmed to be statistically significant (Figure 2).

The weakest activity was observed for the 1% acetic acid extract, which displayed an LD_50_ value of 19.2 mg/mL. Viability values ranged from 49.41% at the highest concentration to 86.05% at the lowest, but all results remained statistically significant (Figure 2).

The relatively low activity of mebendazole observed in this study should be interpreted in the context of the biological characteristics of *Rhabditis* sp. and the in vitro assay conditions. Notably, mebendazole did not reduce nematode viability below 50% at any tested concentration, with viability remaining as high as 86.32% at 22.2 mg/mL, indicating limited susceptibility under short-term exposure.

This may be related to its mechanism of action, which involves binding to β-tubulin and is influenced by drug uptake and species-specific sensitivity. In free-living nematodes such as *Rhabditis* sp., reduced ingestion and physiological differences may further decrease drug effectiveness. Consequently, the higher activity of the tested extracts likely reflects more efficient interactions with the nematodes under the given experimental conditions.

The concentrations applied in the nematicidal assays followed a geometric progression starting from 1.38 mg/mL. For clarity of presentation, selected values were rounded (e.g., 1.38 to 1.4 mg/mL), although this was not performed consistently across all concentrations (e.g., 2.7 mg/mL is shown instead of 2.8 mg/mL, while 5.5, 11.1, and 22.2 mg/mL were retained as calculated). It should be noted that all experiments were conducted using the exact calculated concentrations, and the minor discrepancies observed in the figure labeling are solely due to rounding conventions. These differences do not affect the experimental outcomes or the determination of LD_50_ values.

### 3.4. Antiviral Activity

The antiviral activity of extracts obtained from *S. crispa* fungus was tested against the CVB3 and HSV-1. The MTT method was used to determine non-toxic concentrations of the tested extracts and reference substances for Vero cells, and their antiviral activity was then assessed. The non-toxic concentrations of the water extract (W) and the ethanolic extract (ET60%) were 16 mg/mL and 8 mg/mL, respectively, while those of ribavirin and acyclovir were 0.625 mg/mL and 0.05 mg/mL, respectively.

Initial screening was performed using an inverted optical microscope, and the results were then confirmed using viral titration and molecular methods.

**Figure 5:**
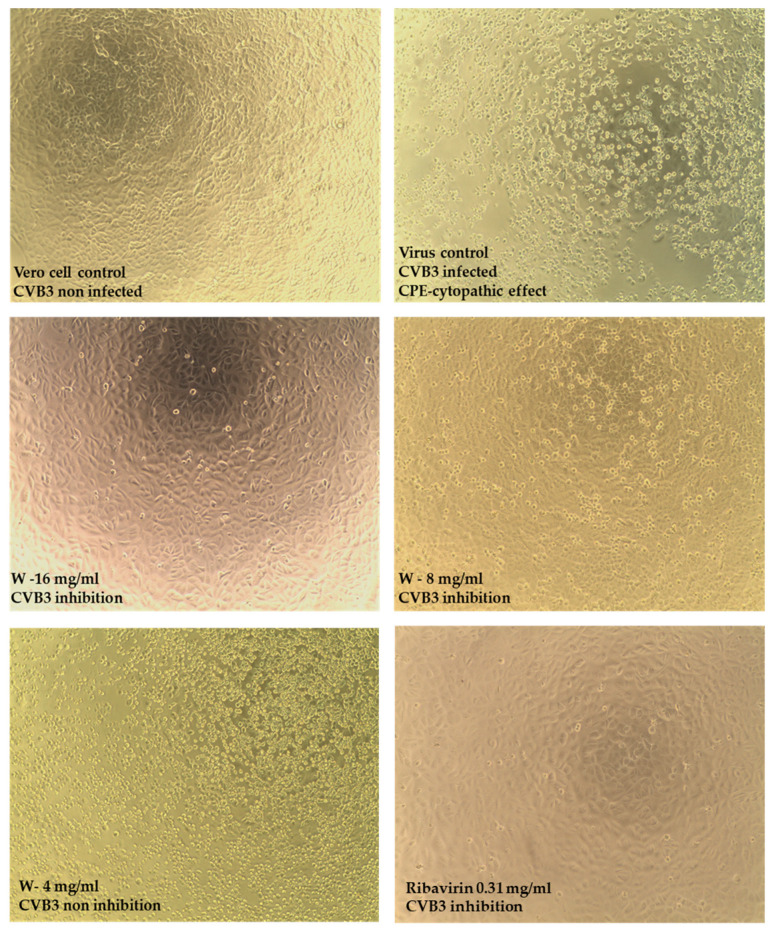
Effect of water (W) extract of *S. crispa* on the development of cytopathic effect (CPE) in Vero cells infected with Coxsackievirus B3 (CVB3). 100× magnification.

**Figure 6:**
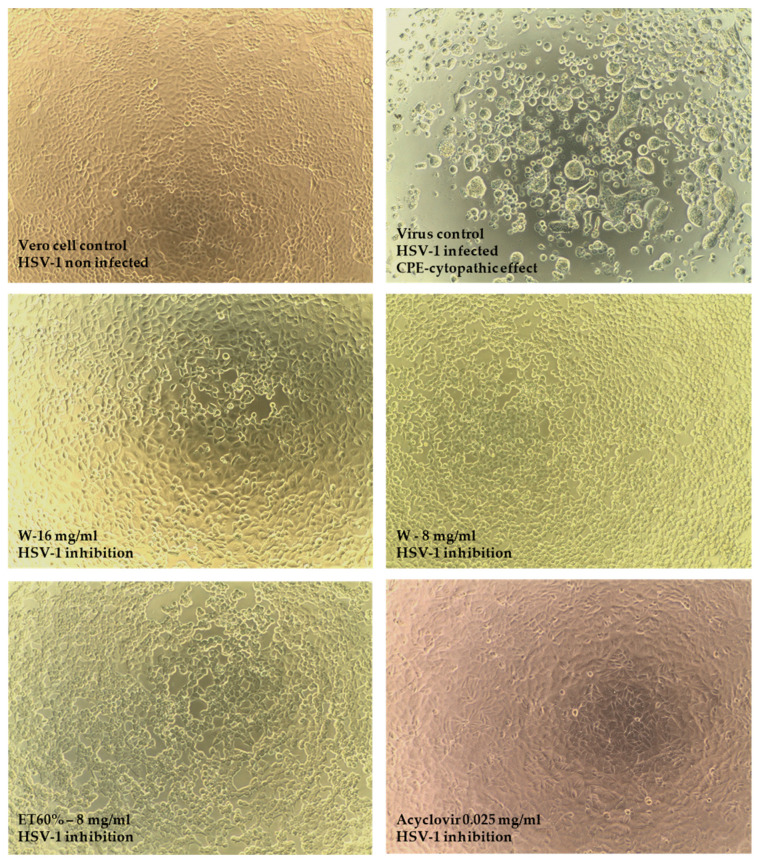
Effect of water (W) and ethanol (ET60%) extracts of *S. crispa* on the development of cytopathic effect (CPE) in Vero cells infected with *Herpes simplex* type 1 (HSV-1). 100× magnification.

In the screening study, CVB3 multiplication was inhibited only by the water (W) extract at concentrations of 8 mg/mL and 16 mg/mL (Figure 5), while the inhibition of HSV-1 multiplication was demonstrated by the following extracts: water (W) at a concentration of 8 mg/mL and 16 mg/mL, and 60% ethanolic extract (ET60%) at a concentration of 8 mg/mL (Figure 6).

The titration method was used to determine the viral titer in samples containing the non-toxic concentration of water (W) extract (16 and 8 mg/mL), ribavirin (RBV) (0.625 and 0.312 mg/mL) for CVB3, acyclovir (0.05 and 0.025 mg/mL) for HSV-1, and a control sample containing only CVB3 or HSV-1. Titration results are presented in CCID_50_/mL (Cell Culture Infectious Dose). The viral titer, expressed as log10, in samples containing the extract, ribavirin, or acyclovir was compared with that in samples without these substances. The water (W) extract at a concentration of 16 mg/mL reduced the viral titer of CVB3 by 2.66 log, or 42% (*p* = 0.043), compared to the virus control. At a concentration of 8 mg/mL, the viral titer was reduced by 1.62 log (26%). On the other hand, ribavirin tested as a reference substance, at a concentration of 0.625 mg/mL and 0.312 mg/mL, reduced the viral titer by 2.77 (*p* = 0.035) and 2.27 log, or by 44% and 36% (Table 4).

**Figure 7:**
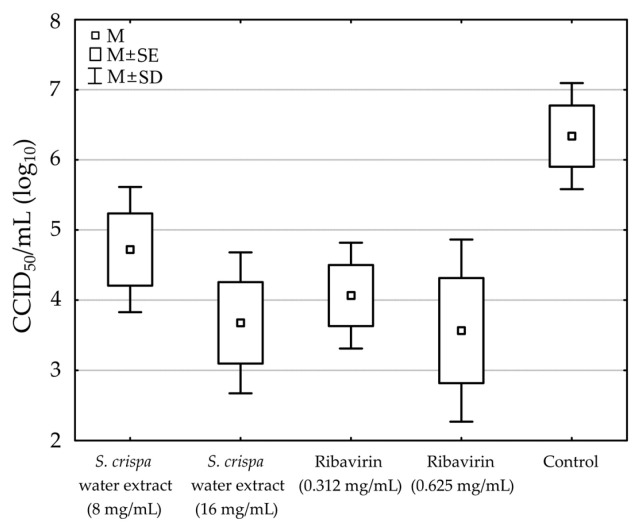
Comparison of CVB3 virus titers (CCID_50_/mL in log_10_) in all samples treated with *S. crispa* water extract (W) or ribavirin or virus control (ANOVA). M—Mean, SD—Standard Deviation, SE—Standard Error of the Mean, F—Analysis of Variance, *p*—*p*-value. Values come from three independent experiments. Statistical significance (*p* < 0.05, F > 2).

Statistically significant differences were observed between the samples [F (4, 10) = 4.191, *p* = 0.030]. Post hoc comparisons using Tukey’s RIR test showed that the mean results for the water extract (16 mg/mL) and ribavirin (0.625 mg/mL) samples were significantly lower than those for the control sample (*p* < 0.05). The remaining differences were not statistically significant (Figure 7).

Although the screening test indicated that the tested extracts inhibited HSV-1 (Figure 6), the titration results did not confirm their antiviral activity. According to RT-qPCR results (Figure 7), the *S. crispa* water extract at 16 mg/mL reduced the CVB3 viral load by 1.07 ± 0.16 log, while the ribavirin (RBV) at 0.625 and 0.312 mg/mL reduced the viral load by 2.8 and 1.92 log, respectively, compared to Vero cell control. Interestingly, the end-point virus titration showed that *S. crispa* water extract at 16 and 8 mg/mL reduced the CVB3 infectious titer by 2.66 and 1.62 log, respectively. The fact that a noticeably higher reduction was observed in the CVB3 viral infectious titer (2.66 log) than in the decrease in viral load (1.07 log) may indicate that viral replication was mainly inhibited during the stages of the replication cycle involving the production of structural proteins or the maturation of viral progeny, rather than during the replication of viral RNA. The inhibition of the later stages of CVB3 replication remains hypothetical and necessitates additional mechanistic studies. Indeed, further studies are planned, including the isolation of bioactive molecules and testing their effects on various steps of the viral replication cycle.

**Figure 8:**
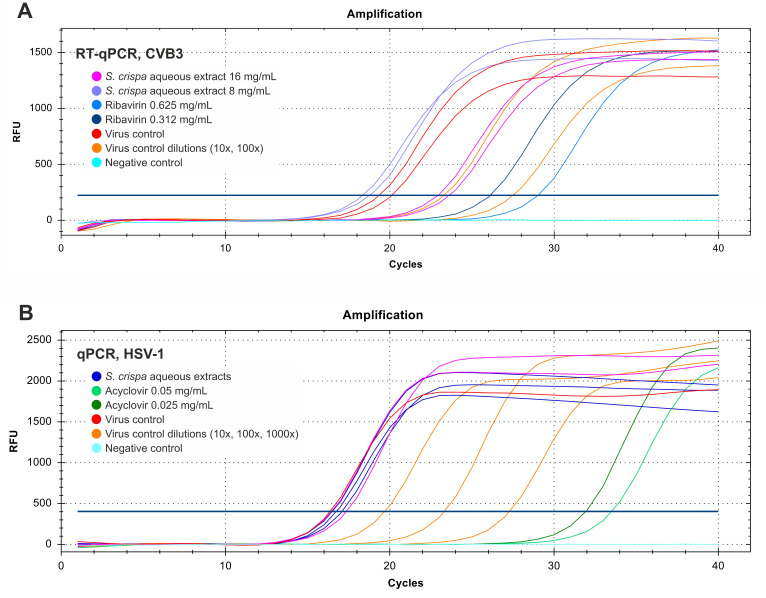
The amplification curves of CVB3 and HSV-1 in samples collected during antiviral experiments. ((**A**)—CVB3 viral load, (**B**)—HSV-1 viral load). CVB3—Coxsackievirus B3, HSV-1—*Herpes simplex* type 1 virus.

None of the tested extracts managed to inhibit the replication of HSV-1, and there were no noticeable effects on the development of HSV-1-induced CPE (cytopathic effect) in virus-infected VERO cells, viral infectious titer, or viral load (Δlog between 0.04 and 0.3). Acyclovir, used as a reference antiherpesviral drug, at 0.05 and 0.025 mg/mL reduced the HSV-1 viral load by 5.16 and 4.7 log, respectively. Detailed RT-qPCR and qPCR results are presented in Figure 8.

An additional factor that should be considered when interpreting the antiviral results is the potential variability in extract solubility. Since all dried extracts were reconstituted in water prior to testing, the evaluated activity reflects only the water-soluble fraction of each extract. Compounds with limited aqueous solubility may not have been fully represented in the assays, which could influence the observed differences in antiviral efficacy between extraction methods.

### 3.5. Antibacterial and Antifungal Activity

The antimicrobial activity of the studied extracts from *S. crispa* was tested towards reference Gram-positive (nine strains) and Gram-negative (five strains) bacteria. Moreover, antifungal effect against yeasts belonging to *Candida* spp. (six strains) was investigated. EUCAST recommends the use of defined ATCC reference strains, including clinically relevant pathogens, for the validation of antimicrobial susceptibility testing to ensure the reproducibility and comparability of results. The ATCC designation provides well-characterized strains with documented pathogenic properties, which is essential for their application in both research and diagnostic settings. Therefore, these strains were selected for the present study.

Among the four *S. crispa* extracts tested, only the 1% acetic acid extract displayed measurable antibacterial activity in our assays. Its minimal concentrations, which inhibited growth of these microorganisms (MIC–Minimum Inhibitory Concentration), ranged from 10 to 20 mg/mL (with a mean activity values 13.33–20 mg/mL). *Micrococcus luteus* was the most susceptible to this extract at MIC = MBC (Minimum Bactericidal Concentration) = 10 mg/mL and MBC/MIC = 1, which indicated its bactericidal effect. In the present study, 1% acetic acid (AA) was used as the extraction solvent, and all extracts were subsequently evaporated to dryness. Therefore, the final preparations did not contain residual 1% AA. Additionally, a control experiment was performed using 1% AA alone under the same conditions, which showed no detectable antimicrobial activity.

None of the tested *S. crispa* extracts exhibited antifungal activity (Table 5).

Overall, Gram-positive bacteria were more susceptible to the tested extracts than the Gram-negative bacteria. This difference may be due to the various structure of their cell walls. It is likely that some active compounds in this extract can more easily break down important bonds in the cell wall structure of Gram-positive bacteria. In turn, the cell wall of Gram-negative bacteria is more developed; this may be why they are more resistant to the studied extract [40].

## 4. Discussion

Numerous chemical constituents have been identified in the fruiting bodies of *Sparassis crispa.* Accumulating evidence indicates that this species exhibits a broad spectrum of biological activities, including health-promoting, antiviral, antibacterial, antioxidant, and immunomodulatory effects [10,17,22]. In addition, the amounts of vitamin D2, exceeding those observed in other mushrooms, vitamin C and E, and a large amount of glucosylceramide and unsaturated fatty acids were found. The majority of the mushroom mass is carbohydrates, with the highest content of β-glucan, which is over 40% in dried basidiomycetes [10]. Sparassol (methyl 2-hydroxy-4-methoxy-6-methylbenzoate) isolated from *S. crispa* has antimicrobial and antifungal properties (against *Candida albicans*). Analysis of the content of minerals and amino acids in the fruiting bodies of *S. crispa* showed significant amounts of potassium, phosphorus, and sodium. Among the amino acids, glutamine and asparagine were the most abundant. The presence of gentisic, gallic, o-coumaric, caffeic, protocatechuic, syringic and *p*-hydroxybenzoic acids, as well as indole, tryptamine, melatonin and ergosterol was also recorded [3,10].

The UHPLC–ESI–MS profiles of *Sparassis crispa* extracts revealed a chemically diverse but only partially resolved metabolite composition. Due to the non-targeted nature of the analysis and the lack of authentic reference standards for most detected compounds, the majority of signals could only be assigned at the level of putative annotation or compound class (MSI Level 2–3). Accordingly, the present dataset should be interpreted as a preliminary metabolic fingerprint, rather than a complete structural characterization.

The detection of nucleoside derivatives, including adenosine and 5′-methylthioadenosine, is consistent with the conserved purine metabolism in basidiomycetes and aligns with the previously reported chemical profiles of edible fungi [8]. These compounds are frequently associated with primary metabolic pathways and have been widely detected in the fungal fruiting bodies.

Phenolic acid-related signals observed in both extracts further indicate the presence of low-molecular-weight aromatic metabolites. However, their exact structural identities could not be confirmed under the applied experimental conditions and therefore remain tentative.

A number of higher-mass features (e.g., *m*/*z* 369, 488, 531, and 611) were classified as conjugated phenolic-like compounds or complex secondary metabolites. While their presence suggests a more complex secondary metabolite pattern, definitive structural elucidation was not possible without MS/MS confirmation and reference standards. Consequently, these compounds should be regarded as metabolite classes, rather than individual molecular entities.

Importantly, features initially resembling plant-derived flavonoids were re-evaluated in the context of fungal metabolism. Given that basidiomycetes are not generally considered to biosynthesize classical flavonoid structures, these signals are more plausibly attributed to structurally related phenolic derivatives or fungal-specific conjugated metabolites. Nevertheless, this interpretation remains tentative and requires further analytical confirmation.

Overall, the obtained results highlight that *S. crispa* contains a metabolically diverse system dominated by nucleoside derivatives and phenolic-type compounds. However, the present UHPLC–ESI–MS analysis should be regarded as a preliminary fingerprinting study, providing a basis for future targeted isolation and structural elucidation [2,41,42,43].

According to current reports, nematicidal activity has also been documented in extracts derived from other fungal taxa. Lee et al. [21] demonstrated that a low-molecular-weight metabolite, sparassol, isolated from culture media of multiple *Sparassis latifolia* strains, exhibited pronounced nematicidal effects against the pine nematode (*Bursaphelenchus xylophilus*). The magnitude of this activity was positively associated with the intracellular concentration of sparassol in the mycelial biomass. Bioassays performed with *B. xylophilus* revealed LC_50_ and LC_95_ values of 84.92 and 132.13 ppm, respectively [21]. Numerous investigations have indicated that species of the genus *Pleurotus* display particularly pronounced nematicidal properties. Antiparasitic effects have been documented in extracts and fractions derived from both fruiting bodies and mycelial cultures. The earliest evaluations of the in vitro nematicidal activity of an aqueous extract of *Pleurotus ostreatus*, conducted approximately two decades ago, led to the identification of trans-2-decenedioic acid as the principal active compound. Within one hour of exposure, this metabolite reduced the viability of *Panagrellus redivivus* by 95%, thereby confirming the potency of the aqueous extract [44].

Giacometi et al. [45] investigated the nematicidal potential of *Pleurotus* spp. against intestinal nematodes, with particular emphasis on cyathostomine parasites of horses, which are associated with substantial declines in host health and condition. In vitro assays assessing the effects of aqueous extracts of *P. ostreatus*, *P. florida*, and *P. djamor* on cyathostomine eggs revealed that *P. florida* exhibited the most marked inhibition of larval hatching. At the highest extract concentration (10%), egg hatching was suppressed by 92.19%, indicating its strong potential as an alternative strategy for nematode control. In contrast, *P. ostreatus* and *P. djamor* displayed comparatively weaker inhibitory effects, achieving 55.46% and 23.67% inhibition, respectively, at the same concentration.

Castañeda-Ramírez et al. [46] evaluated the nematicidal activity of *P. florida* using ethanol–water (1:1) extracts at concentrations of 10 mg/mL and 50 mg/mL, as well as a 100 mg/mL preparation diluted in distilled water. The authors described dose-dependent effects, as a result of which exposed nematodes gradually lost their motility, became paralyzed, and ultimately died. The study showed that *P. florida* extracts, thanks to their rich composition of amino acids, carbohydrates, glycosides, flavonoids, and tannins, exhibited greater antiparasitic efficacy than conventional therapeutic agents. Consistent with these results, our own research showed that an ethanol extract derived from the fruiting bodies of *S. crispa* demonstrated the most potent nematicidal activity among the extracts tested.

Further analyses of *Pleurotus pulmonarius* and *Hericium coralloides*, both classified within the *Basidiomycetes*, demonstrated nematicidal activity against *Caenorhabditis elegans*. Six antiparasitic compounds were isolated from fungal cultures, with linoleic acid and S-coriolic acid displaying the greatest potency, reflected in LD_50_ values ranging from 5 to 10 ppm [47].

Furthermore, investigations employing extracts derived from the mycelium of *P. pulmonarius* demonstrated pronounced immobilizing activity against pre-infective larvae of the nematode species *Ostertagia ostertagi*, *Cooperia oncophora*, *Oesophagostomum quadrispinulatum*, and *Cyathostoma* sp. The *P. pulmonarius* extracts exhibited significantly stronger effects on pre-infective stages (70%) than on infective larvae (30%) [48]. Consistent with these findings, Hugo et al. [49] confirmed the nematicidal activity of *P. ostreatus* and its isolated proteases toward *Panagrellus* sp. larvae, reporting a reduction in larval viability to 5% following 72 h of incubation [49]. Similarly, an aqueous extract of *P. eryngii* markedly decreased the number of viable *Panagrellus* sp. individuals after 24 h and 48 h to 40% and 10%, respectively, although this activity was not attributable to protease action [50].

Chuixu et al. [51] further assessed the nematicidal properties of *Stropharia* sp. mycelium against *Panagrellus redivivus*, demonstrating 100% mortality after 24 h of exposure to an aqueous extract. In addition, acetone and methanol extracts obtained from *P. ostreatus* fruiting bodies elicited substantial mortality in *P. redivivus* within only 1 h, reaching 80% and 92% mortality, respectively [52]. Complementing these observations, Soares and colleagues [53] demonstrated that extracts derived from the mycelium of *Hypsizygus marmoreus* reduced *P. redivivus* viability by up to 52%, attributable to protease activity present in the extract [53].

In our study, nematodes of the genus *Rhabditis* were selected for nematicidal activity assessment. The used nematode model was developed at the Department of Biology and Genetics of the Medical University of Lublin, Poland. In 2017, the model obtained patent protection covering the method for culturing *Rhabditis* spp. and determining their nematicidal activity (P.421846; Authors: A. Bogucka-Kocka, P. Kołodziej). Currently, this model is unique on a global scale. Previously, anthelmintic activity tests were performed using earthworm or *Caenorhabditis elegans* models; however, these do not fully reflect the parasitic nature of the organisms under study.

The nematicidal activity of *S. crispa* extracts was confirmed across all tested extract types, with statistically significant reductions in *Rhabditis* sp. viability after 24 h of exposure (Figure 2). Among the evaluated preparations, the 60% ethanol extract exhibited the strongest activity and the lowest LD_50_ value, followed by the MAW and aqueous extracts. The 1% acetic acid extract showed only weak activity (Table 2). Observed morphological changes and impaired motility further corroborated the toxic effects of the most active extracts (Figure 3 and Figure 4). Comparison with the existing literature demonstrates that the nematicidal properties of *S. crispa* are consistent with those described for other *Basidiomycetes*, reinforcing the potential relevance of fungal metabolites in the development of alternative antiparasitic strategies.

Within this broader context, our findings establish *S. crispa* as another mushroom with significant antiparasitic potential. The fact that all *S. crispa* extracts were active suggests that multiple compound classes (polar and non-polar) may contribute to its nematicidal properties. Ethanol appears to extract the most potent fraction, possibly enriching relatively non-polar bioactives such as terpenoids or benzoate derivatives (e.g., sparassol) that can penetrate and disrupt nematode tissues. The observable paralysis and reduced motility of nematodes even at sub-lethal concentrations of the *S. crispa* ethanol extract further indicate neurotoxic or neuromuscular effects, reminiscent of the action of fatty acids and proteases from other fungi. Importantly, the nematicidal activity of *S. crispa* in our study was achieved at concentrations that are within the same order of magnitude as those reported for pure fungal compounds, highlighting the remarkable efficacy of these extracts. Future work should focus on isolating and identifying the specific nematicidal agents in *S. crispa*. Candidates of interest include sparassol and related benzoic acid derivatives, as well as unsaturated fatty acids or other small molecules that might be present in the ethanol extract. Clarifying the mechanism of action (e.g., whether these metabolites cause oxidative stress, neuromuscular blockade, or metabolic disruption in nematodes) will also be valuable. Overall, the demonstration of antiparasitic activity in *S. crispa* supports its development as a source of novel anthelmintic agents, which could be beneficial given rising resistance to existing antiparasitic drugs and the need for new, sustainable approaches in parasite control.

The pronounced nematicidal activity demonstrated in the present study underscores the biotechnological potential of *S. crispa* as a source of natural compounds with applications extending beyond pharmacology toward food and agricultural systems. In the context of the growing interest in mushrooms as sustainable bioresources, the identification of nematicidal constituents in *S. crispa* is particularly relevant for integrated crop protection strategies and the development of environmentally compatible plant health products. Natural metabolites derived from edible fungi may offer biodegradable and low-residue alternatives to synthetic nematicides, aligning with current priorities in sustainable food production and agroecology.

Modern medicine does not have an adequate number of effective antiviral drugs, and currently used drugs are often not very specific to viruses. They usually have a narrow spectrum of activity and several side effects for the patient. Another problem is the phenomenon of virus resistance to currently used drugs. Therefore, there is a constant need to search for new therapeutics. Numerous studies worldwide indicate natural raw materials, including plants and fungi, as an excellent potential source of new drugs.

Many researchers have evaluated mushrooms’ medicinal properties, and other species’ pharmacological properties are being discovered. An important issue is identifying the metabolites and compounds present in extracts which are responsible for their action, chemical characteristics, and mechanism of action. There is also a need for a complete understanding of both their individual and synergistic actions [54].

Joining the search for new antiviral drugs of natural origin, we investigated the potential antiviral activity of extracts from the *S. crispa* mushroom. The antiviral activity of three extracts from the fungus *S. crispa* against the viruses CVB3 and HSV-1 was studied. Concentrations of the tested extracts below 16 mg/mL showed no toxicity in Vero cells. Two extracts (water and ethanolic) showed antiviral activity during the screening study. In particular, the water extract used at a concentration of 16 mg/mL significantly reduced the infectivity of CVB3, as demonstrated in an in vitro study using the viral titer reduction method. The obtained results indicate that it contains substances with antiviral activity, which may be a promising source of new antiviral drugs. However, the concentration at which the antiviral activity was observed remains relatively high. Future studies will determine whether isolated bioactive fractions or molecules exhibit antiviral activity at lower concentrations. The remaining extracts obtained from *S. crispa* did not show significant antiviral activity.

Fungi produce different classes of secondary metabolites that could be used as antiviral agents in the future. The study conducted by Boudagga [55] aimed to determine the antiviral activity against HSV-2 and CVB3 of methanolic extracts obtained from two edible mushrooms, *Boletus bellinii* and *Boletus subtomentosus*, collected in the northern forests of Tunisia. In vitro studies on Vero cells showed that the tested methanolic extracts of both species were not cytotoxic at doses > 1 mg/mL and showed significant virus inhibition with IC_50_ of 3.60 µg/mL and 35.70 µg/mL for *B. bellinii* and 5.67 µg/mL and 56.88 µg/mL for *B. subtomentosus*, against HSV-2 and CVB3, respectively. Replication of both viruses was inhibited at early stages. Inonotusin A, responsible for these effects, was isolated from extracts of the *Boletus* genus, which was first described in the cited work [55].

The *S. crispa* water extract did not exert significant antiviral effects against CVB3 or HSV-1. However, for CVB3, a noticeable reduction in the CVB3 infectious titer was observed. Given the lower impact on the CVB3 virus, we hypothesized that *S. crispa* water extract partially inhibited the later stages of the CVB3 replication cycle. We previously reported a similar effect of *Spathodea campanulata* leaf methanolic extract against HSV-1, where bioactive compounds were shown in silico to inhibit viral proteases [56].

The next part of our research focused on determining the extracts’ antiviral activity against the HSV-1 virus. The screening study showed that the water extract (W) and ethanol extract (ET60%) demonstrated antiviral activity. However, it was not possible to confirm this using the titration method. After calculating the viral titer, it turned out that none of the tested extracts reduced the viral titer in infected cells. The literature provides many examples of studies confirming that searching for compounds with antiviral activity, also directed against the HSV virus, in the fungi kingdom is advisable. A few experiments prove that individual compounds or extracts from the fungi world can be excellent research objects in discovering new effective antiviral drugs. *Inonotus obliquus*, also known as Chaga, is a medicinal mushroom which has been used for therapeutic purposes since the 16th century. Various extracts and compounds isolated from *I. obliquus* possess promising anti-inflammatory, antioxidant, antibacterial, and antiviral properties [57]. Extracts of *I. obliquus* also exhibit anti-herpetic activity on HSV-infected Vero cells. The anti-HSV mechanism was found to be effective against the early stage of viral infection through inhibition of viral-induced membrane fusion. Therefore, the aqueous extract from *I. obliquus* could effectively prevent HSV-1 entry by acting on viral glycoproteins, leading to the prevention of membrane fusion, which is different from nucleoside analog antiherpetics [58].

Another example of the antiviral activity of fungi is the study of compounds formed during salt fermentation of a marine fungus of the genus *Scytalidium*. In vitro studies have shown that lipophilic, neutrally charged peptides called halovirs A–E, formed in this way, directly inactivate HSV 1 and 2. The mechanism of action of halovirs could be used to prevent HSV transmission by developing a topical antiviral agent that incorporates these peptides [59].

In turn, studies conducted on fungi from the *Grifolaceae* family have shown the antiviral activity of the *Grifola frondosa* extract. A new antiviral protein called GFAHP (*Grifola frondosa* anti-herpes simplex virus protein) was obtained from the extract of the fruiting bodies of this fungus, which showed the ability to inhibit the replication of the HSV-1 in an in vitro study. In studies conducted on mice, higher concentrations of GFAHP caused a significant reduction in the severity of symptoms caused by HSV-1, such as blepharitis, neovascularization, and inflammation of the corneal stroma. Studies have shown that GFAHP acts directly on the HSV-1 virus, leading to its inactivation. Additionally, this protein can inhibit the penetration of the virus into Vero cells, which were used in the present study. The antiviral substance GFAHP was found to be effective against the HSV-1 virus both in vitro and in vivo [60].

The antiviral activity of mushrooms was confirmed by Wang J et al. [61], who tested aqueous extracts of 16 species, including *S. crispa*, for human HIV-1 reverse transcriptase inhibitory activity. The HIV-1 RT inhibition ratio of samples from examined medicinal mushrooms ranged from 9.2% to 97.6% when the extracts were tested at a concentration of 1 mg/mL. The inhibition ratio of the following five extracts was above 50%: *Lactarius camphorates*, *Rametes suaveloens*, *Pleurotus sajor-caju*, *Pleurotus pulmonarius*, and *Russula paludosa*. Extract from *S. crispa* demonstrated an inhibition ratio of 70.3%. Inhibition of the RT enzyme is a key element in antiretroviral therapies directed against HIV, which opens potential perspectives for the use of this extract in the treatment of AIDS [61].

Considering our findings alongside the literature, it appears that *S. crispa* may harbor antiviral constituents that are effective under certain conditions or against specific viruses. The moderate activity against CVB3 points to one or more water-soluble compounds that can impair enterovirus infectivity—these might include polysaccharides or polyphenols known for antiviral effects via immunomodulation or direct virucidal action. The lack of an effect on HSV-1, a DNA virus with a different replication strategy and a protective envelope, indicates that *S. crispa* extracts either do not penetrate or affect the viral envelope or that the active compounds are virus-specific. It is possible that optimizing the extract (e.g., concentrating certain fractions) or testing additional strains could reveal anti-herpetic activity not seen in our broad extract approach. Moreover, guided isolation using the CVB3 assay as a readout could lead to the identification of the active principle in the water extract. If isolated, such a compound could be studied in more detail for its mechanism—for instance, whether it inhibits viral protease or capsid functions, as has been reported for plant-derived antivirals. In summary, the aqueous extract’s effect against CVB3, though partial, is a proof-of-concept that *S. crispa* produces antiviral agent(s). Given the continual emergence of drug-resistant viruses and limited antiviral options for many infections, *S. crispa* merits further exploration as a natural source of antivirals. Future studies should extend screening to other virus types (including influenza, hepatitis, or coronaviruses) and isolate the bioactive molecules for structure elucidation. Additionally, synergistic effects among *S. crispa* metabolites (or with standard antivirals) could be explored to see if a combination yields stronger antiviral efficacy than individual components.

According to research conducted by Dyakow et al. [62], *S. crispa* possess antibacterial activity towards many species including six Gram-positive strains: *Bacillus mycoides*, *Bacillus subtilis*, *Bacillus pumilis*, methicillin-resistant *Staphylococcus aureus* (MRSA), *Micrococcus luteus* and *Leuconostoc mesenteroides* and one Gram-negative bacteria *Comamonas terrigena*. In turn, Lee et al. [63] reported antimicrobial effect of extracts from *S. crispa* towards selected bacteria associated with food poisoning: *Bacillus cereus*, *Listeria monocytogenes*, *Salmonella typhimurium*, and *Staphylococcus aureus*. The antibacterial effect persisted even after heating and treatment with alcalase, indicating its potential use as a natural preservative in the food industry.

Antimicrobial activity of the mushroom sample was also tested by other authors [64] against some bacteria using the agar well diffusion method. Their results showed maximum antibacterial activity of *S. crispa* ethanolic extract, with an inhibition zone of 19.66 ± 0.88 mm against *Escherichia coli* compared to other bacterial strains. The next researchers [65] conducted studies to investigate the possibilities of applying cosmetic materials to extracts from *S. crispa*. These extracts exhibited antimicrobial effects against *Straphylococcus epidermidis*, *S. aureus* and *E. coli* using the paper disc method. These results suggest that extracts from *S. crispa* may also be valuable for potential cosmetic formulations.

In the course of screening for anti-MRSA activity, the authors of [66] reported the antibacterial effects of an extract from *S. crispa* and isolated two compounds as the active principles. Identification of these components was based on the interpretation of data obtained using nuclear magnetic resonance (NMR) and mass spectrometry (MS). The presence of the following compounds: chalcones, xanthoangelol and 4-hydroxyderricin was confirmed. Pharmacologically, chalcones are important compounds with a broad spectrum of biological activities, including antibacterial, antifungal, anticancer, and anti-inflammatory properties [66].

Hayashi et al. [66] also isolated these compounds from some extracts of *S. crispa* and showed their potential anti-staphylococcal activity. The results exhibited that xanthoangelol and 4-hydroxyderricin were active against MRSA and inhibited their growth at MIC values of 2 and 0.25 mM, respectively. In turn, Woodward et al. [4] identified three metabolites with antifungal effect from submerged cultures of *S. crispa* in 2% malt broth. One of these compounds was sparassol (methyl-2-hydroxy-4-methoxy-6-methylbenzoate). The remaining two, named ScI and ScII, showed significantly greater antifungal activity than sparassol towards *Cladosporium cucumerinum*, and were characterized as methyl-2,4-dihydroxy-6-methylbenzoate (methyl orsellinate) and an incompletely determined methyl-dihydroxy-methoxy-methylbenzoate, respectively. These compounds were also reported from the decayed wood of trees infected by *S. crispa* [66]. Another scientist [10] also observed that *S. crispa* produces antibiotic substances and added that inhibition of *Bacillus subtilis* growth on agar media is caused by sparassol.

The antibacterial mechanism of *S. crispa* polysaccharides (SCPs) has been demonstrated in some studies [23]. These SCPs consisted of fucose, glucose, and galactose in a molar ratio of 0.043:0.652:0.305. The results indicated better inhibition of *S. aureus* growth than *E. coli* by SCPs and bacterial damage visible under scanning electron microscopy. HPLC-Q-TOF/MS analysis showed that SCPs disrupted the glycolysis and tricarboxylic acid cycle pathways in *S. aureus*. Significant changes were observed in fructose-1,6-diphosphate, 1,3-diphosphoglycerol, succinate, and oxaloacetate, as well as a decrease in ATP in cells, which may indicate that SCPs exert antibacterial effects by inducing disturbances in catabolism and energy metabolism. This research confirmed the antibacterial properties of SCPs. Another study [3] also confirmed the antibacterial activity of *S. crispa* against *S. aureus* and *E. coli*. Moreover, Jang et al. [65] observed the growth inhibition of *Candida albicans* by *S. crispa*.

In our study, the tested microorganisms comprised both established pathogens and representatives of the normal microbiota. The pathogenic group included *Pseudomonas aeruginosa*, *Klebsiella pneumoniae*, *Salmonella* spp., and *Staphylococcus aureus*, which are associated with a broad spectrum of infections, including those of the respiratory tract, bloodstream, urinary tract, skin, and gastrointestinal system. In addition, selected commensal organisms with opportunistic potential were included, such as *Staphylococcus epidermidis*, *Candida* spp., and certain strains of *Escherichia coli*. Although these microorganisms are part of the normal microbiome, they may cause infection under predisposing conditions, including immunosuppression, surgical intervention, catheterization, or prior antibiotic therapy. These microorganisms were selected to reflect both the diversity of clinically relevant infections and the increasing prevalence of antimicrobial resistance, which contributes to significant therapeutic challenges.

Our study indicates that *S. crispa* exhibits antimicrobial effects; however, the results should be interpreted with caution. The antibacterial and antifungal activities of the crude extracts were moderate, as reflected by the relatively high MIC values, suggesting limited potency in their current form. The observed bioactivity therefore appears selective rather than broad-spectrum.

The bactericidal effect against *M. luteus* and inhibitory activity toward *E. coli* and *S. aureus* nonetheless point to the presence of compounds capable of affecting microbial viability. Previous metabolomic analyses by Lan et al. [23] have suggested that polysaccharide fractions from *S. crispa* may modulate bacterial metabolism rather than exert direct antimicrobial effects. This supports the hypothesis that the overall activity may arise from multiple mechanisms, including direct effects of low-molecular-weight metabolites and indirect modulation mediated by polysaccharides such as β-glucans.

Overall, the findings indicate that *S. crispa* constitutes a source of bioactive compounds with measurable, albeit limited and selective, antimicrobial and antiviral properties. Further research is required to identify the active components, clarify their mechanisms of action, and assess their relevance under conditions more closely reflecting potential applications.

## 5. Limitations of the Study

Although the present study provides new insight into the chemical composition and biological activity of *Sparassis crispa* extracts, several limitations should be acknowledged. First, chemical characterization was performed using a non-targeted UHPLC–ESI–MS approach, which allowed for metabolic fingerprinting rather than full structural elucidation. Consequently, most detected features were assigned at the level of putative annotation or compound class (MSI Level 2–3), and only a limited number of metabolites could be tentatively identified based on literature comparison and available spectral data. The absence of authentic reference standards for the majority of compounds precluded definitive (Level 1) confirmation.

Second, chromatographic profiling was restricted to the water and 60% ethanolic extracts, which represented the most biologically active fractions in the preliminary screening. While this approach allowed the prioritization of chemically relevant fractions, it did not provide complete metabolomic coverage of all tested extracts, and therefore limited direct chemical–bioactivity comparison across all extraction systems.

Third, no quantitative determination of marker compounds was performed. The study is therefore qualitative in nature, and the relative abundance of individual metabolites cannot be reliably correlated with the observed biological effects. Future studies employing targeted LC–MS/MS quantification would be necessary to establish dose–response relationships and identify bioactive constituents more precisely.

Finally, although MS/MS fragmentation and UV–Vis data supported the proposed annotations, the lack of high-resolution mass spectrometry (HRMS) for all features limits the certainty of molecular formula assignment. Consequently, the chemical profiles presented herein should be interpreted as preliminary metabolic fingerprints rather than fully resolved phytochemical (or mycochemical) compositions.

Overall, these limitations highlight the exploratory nature of this study and indicate that further targeted and quantitative analyses are required to fully elucidate the bioactive constituents of *S. crispa* extracts.

## 6. Conclusions

The present study expands the functional characterization of *Sparassis crispa* as an edible mushroom with significant bioactive properties. Distinct solvent fractions of the fruiting bodies exhibited nematicidal, antiviral, and antibacterial activities, highlighting the chemical diversity of metabolites present in this species. These findings support the concept of edible fungi as reservoirs of multifunctional compounds relevant to food innovation, sustainable agriculture, and health-oriented product development.

Future research should prioritize the isolation and chemical identification of active constituents, elucidation of their mechanisms of action, and evaluation of their stability and safety in food matrices and in vivo models. Integrative approaches that combine metabolomics, bioassay-guided fractionation, and systems biology are essential for fully elucidating the functional potential of *S. crispa*. By bridging food science, microbiology, and natural product chemistry, such studies may contribute to the development of next-generation functional foods and bio-based solutions derived from edible mushrooms.

## 7. Patent

On the basis of the research results presented in the work, a patent was granted for the invention by the Patent Office of the Republic of Poland (No. 248483).

## Figures and Tables

**Table 1:** Microorganisms used in the study.

Type	Strain	Collection ID
Gram-positive bacteria	*Staphylococcus aureus* (Methicillin Resistant *S. aureus*—MRSA)	ATCC 43300
	*Staphylococcus aureus* (Methicillin Susceptible *S. aureus*—MSSA)	ATCC 29213
	*Staphylococcus aureus* (MSSA)	ATCC 6538
	*Staphylococcus aureus* (MSSA)	ATCC 25923
	*Staphylococcus epidermidis*	ATCC 12228
	*Enterococcus faecalis*	ATCC 29212
	*Micrococcus luteus*	ATCC 10240
	*Bacillus subtilis*	ATCC 6633
	*Bacillus cereus*	ATCC 10876
Gram-negative bacteria	*Bordetella bronchiseptica*	ATCC 4617
	*Escherichia coli*	ATCC 25922
	*Klebsiella pneumoniae*	ATCC 13883
	*Proteus mirabilis*	ATCC 12453
	*Salmonella* Typhimurium	ATCC 14028
	*Pseudomonas aeruginosa*	ATCC 9027
Yeasts	*Candida albicans*	ATCC 10231
	*Candida parapsilosis*	ATCC 22019
	*Candida glabrata*	ATCC 90030
	*Candida krusei*	ATCC 14243
	*Candida tropicalis*	ATCC 1369
	*Candida auris*	CDC B11903

**Table 2:** The extraction efficiency of *S. crispa* dry mass of fruiting bodies using four different solvents.

Solvent	Extraction Efficiency (g from 10 g Dry Mass)
Ethanol 60% (ET60%)	3.769
Methanol-Acetone-Water (MAW)	3.345
Water (W)	4.281
1% Acetic Acid (1% AA)	4.131

**Table 4:** Antiviral activity of *S. crispa* against CVB3.

Compounds	Concentration [mg/mL]	[CCID_50_/mL] *			Results of Statistical Testing
		M	SD	SE	
*S. crispa* water extract (W)	8	4.72 ^ab^	0.89	0.52	F (4, 10) = 4.191, *p* = 0.030 *p* = 0.043 (W 16 mg/mL vs. Control) *p* = 0.035 (ribavirin 0.625 mg/mL vs. Control)
	16	3.68 ^a^	1.00	0.58	
Ribavirin	0.312	4.06 ^ab^	0.75	0.43	
	0.625	3.57 ^a^	1.30	0.75	
Control	0	6.34 ^b^	0.76	0.44	

* The virus titers are shown in log CCID_50_. a, b—means followed by the same letter are not significantly different, CCID_50_—50% Cell Culture Infectious Dose, M—Mean, SD—Standard Deviation, SE—Standard Error of the Mean, F—Analysis of Variance, *p*—*p*-value (statistical significance: *p* < 0.05, F > 2). Values come from three independent experiments.

**Table 5:** The antimicrobial activity of the 1% acetic acid extract from *Sparassis crispa* (1% AA) expressed as MIC (Minimum Inhibitory Concentration), MBC (Minimal Bactericidal Concentration) or MFC (Minimum Fungicidal Concentration) [mg/mL], and MBC/MIC or MFC/MIC values against the reference strains of bacteria and fungi.

Strain		1% AA	CIP/VA/NY
**Gram-positive** **bacteria**	*Staphylococcus aureus* ATCC 43300 (MRSA)	16.67 (+/−5.77), (>20) {>1}	0.24 (0.24) {1}
	*Staphylococcus aureus* ATCC 6538	16.67 (+/−5.77), (>20) {>1}	0.24 (0.24) {1}
	*Staphylococcus aureus* ATCC 25923	20 (+/−0), (>20) {>1}	0.48 (0.48) {1}
	*Staphylococcus aureus* ATCC 29213	16.67 (+/−5.77), (>20) {>1}	0.48 (0.48) {1}
	*Staphylococcus epidermidis* ATCC 12228	13.33 (+/−5.77), (>20) {>1}	0.12 (0.12) {1}
	*Enterococcus faecalis* ATCC 29212	20 (+/−0), (>20) {>1}	0.98 * (1.95) {2}
	*Micrococcus luteus* ATCC 10240	10 (+/−0), (10) {1}	0.98 (1.95) {2}
	*Bacillus subtilis* ATCC 10876	13.33 (+/−5.77), (20) {1}	0.03 (0.03) {1}
	*Bacillus cereus* ATCC 6633	20 (+/−0), (>20) {>1}	0.06 (0.12) {2}
**Gram-negative** **bacteria**	*Bordetella bronchiseptica* ATCC 4617	16.67 (+/−5.77), (>20) (>1)	0.98 (0.98) {1}
	*Klebsiella pneumoniae* ATCC 13883	20 (+/−0), (20) {1}	0.12 (0.24) {2}
	*Proteus mirabilis* ATCC 12453	20 (+/−0), (20) {1}	0.03 (0.03) {1}
	*Salmonella typhimurium* ATCC 14028	20 (+/−0), (>20) (>1)	0.06 (0.06) {1}
	*Escherichia coli* ATCC 25922	16.67 (+/−5.77), (>20) {>1}	0.004 (0.008) {2}
	*Pseudomonas aeruginosa* ATCC 9027	20 (+/−0), (>20) {>1}	0.48 (0.98) {2}
**Yeasts**	*Candida albicans* ATCC 10231	-	0.48 ** (0.48) {1}
	*Candida parapsilosis* ATCC 22019	-	0.24 ** (0.48) {2}
	*Candida glabrata* ATCC 90030	-	0.24 ** (0.48) {2}
	*Candida krusei* ATCC 14243	-	0.24 ** (0.24) {1}
	*Candida tropicalis* ATCC 1369	-	0.24 ** (0.48) {2}
	*Candida auris* CDC B11903	-	0.48 ** (0.48) {1}

The standard antimicrobial drugs used as positive controls: ciprofloxacin (CIP) for bacteria (except enterococci), vancomycin (VA *) for enterococci and nystatin (NY **) for fungi [µg/mL]. Data are represented as mean, standard deviation (+/−SD), MBC or MFC values in parentheses, and MBC/MIC or MFC/MIC values in curly braces.

## Data Availability

The original contributions presented in this study are included in the article/Supplementary Materials. Further inquiries can be directed to the corresponding author.

## Supplementary Materials

The following supporting information can be downloaded at: https://www.mdpi.com/article/10.3390/foods15091559/s1, Figure S1: The UHPLC analysis of the chemical composition of water and 60% ethanolic extracts from *S. crispa*.

## Abbreviations

The following abbreviations are used in this manuscript:

AA	Acetic Acid Extract
AIDS	Acquired Immune Deficiency Syndrome
ATCC	American Type Culture Collection
CCID	Cell Culture Infectious Dose
CIP	Ciprofloxacin
CPE	Cytopathic effect
CVB3	Coxsackievirus B3
DAD	Diode Array Detector
DMEM	Dulbecco’s Modified Eagle Medium
EUCAST	European Committee for Antimicrobial Susceptibility Testing
GFAHP	Grifola frondosa anti-herpes simplex virus protein
HIV	Human Immunodeficiency Virus
HPLC-Q-TOF/MS	High-Performance Liquid Chromatography coupled with Quadrupole Time-of-Flight Mass Spectrometry
HSV-1	*Herpes simplex* virus type 1
IC	Inhibitory Concentration
LD	Lethal Dose
MAW	Methanol:Acetone:Water Extract
MBC	Minimum Bactericidal Concentration
MFC	Minimum Fungicidal Concentration
MIC	Minimum Inhibitory Concentration
MRSA	Methicillin-resistant *Staphylococcus aureus*
MS	Mass spectrometry
MTT	Methyl Thiazolyl Tetrazolium
NaCl	Sodium Chloride
NMR	Nuclear Magnetic Resonance
NY	Nystatin
RBV	Ribavirin
RT-qPCR	Reverse Transcription quantitative Polymerase Chain Reaction
SCPs	*S. crispa* polysaccharides
UHPLC-MS/MS	Ultra-High Performance Liquid Chromatography-Tandem Mass Spectrometry
UV-Vis	Ultraviolet-Visible Spectroscopy
VA	Vancomycin
W	Water Extract
