# Supplementary material for: Extracts from the Edible Mushroom Sparassis crispa: Nematicidal, Antimicrobial, and Antiviral Properties Supporting Its Functional Food Potential
Source: Foods. 2026 May 1;15(9):1559. doi: 10.3390/foods15091559 (PMC13163984; doi:10.3390/foods15091559)
Supplement: Supplementary file 1 [file foods-15-01559-s001.zip › foods-4251740-supplementary.pdf]

Supplementary Material

Extracts from the Edible Mushroom *Sparassis crispa*: Nematicidal, Antimicrobial, and Antiviral Properties Supporting Its Functional Food Potential

Marta Ziaja-Sołtys [1,] *, Barbara Rajtar [2], Łukasz Świątek [2], Anna Biernasiuk [3], Katarzyna Dos Santos Szewczyk [4], Sebastian Granica [5], Andrzej Parzonko [5], Daniel Zalewski [1], Łucja Smolarska [1], Sebastian Skowron [2], Anna Bogucka-Kocka[1]

[1] Chair and Department of Biology and Genetics, Medical University of Lublin, Chodźki 4a, 20-093 Lublin, Poland; daniel.zalewski@umlub.edu.pl (D.Z.)
[2] Department of Virology with Viral Diagnostics Laboratory, Medical University of Lublin, Chodźki 1, 20-093 Lublin, Poland; lukasz.swiatek@umlub.edu.pl (Ł.Ś.)
[3] Chair and Department of Pharmaceutical Microbiology, Medical University of Lublin, Chodźki 1, 20-093 Lublin, Poland
[4] Department of Pharmaceutical Botany, Chair of Pharmacognosy and Pharmaceutical Botany, Medical University of Lublin, Chodźki 1, 20-093 Lublin, Poland
[5] Department of Pharmaceutical Biology, Faculty of Pharmacy, Medical University of Warsaw, 1 Banacha Street, 02-097 Warsaw, Poland; andrzej.parzonko@wum.edu.pl (A.P.)

* Correspondence: Marta Ziaja-Sołtys, marta.ziaja-soltys@umlub.edu.pl

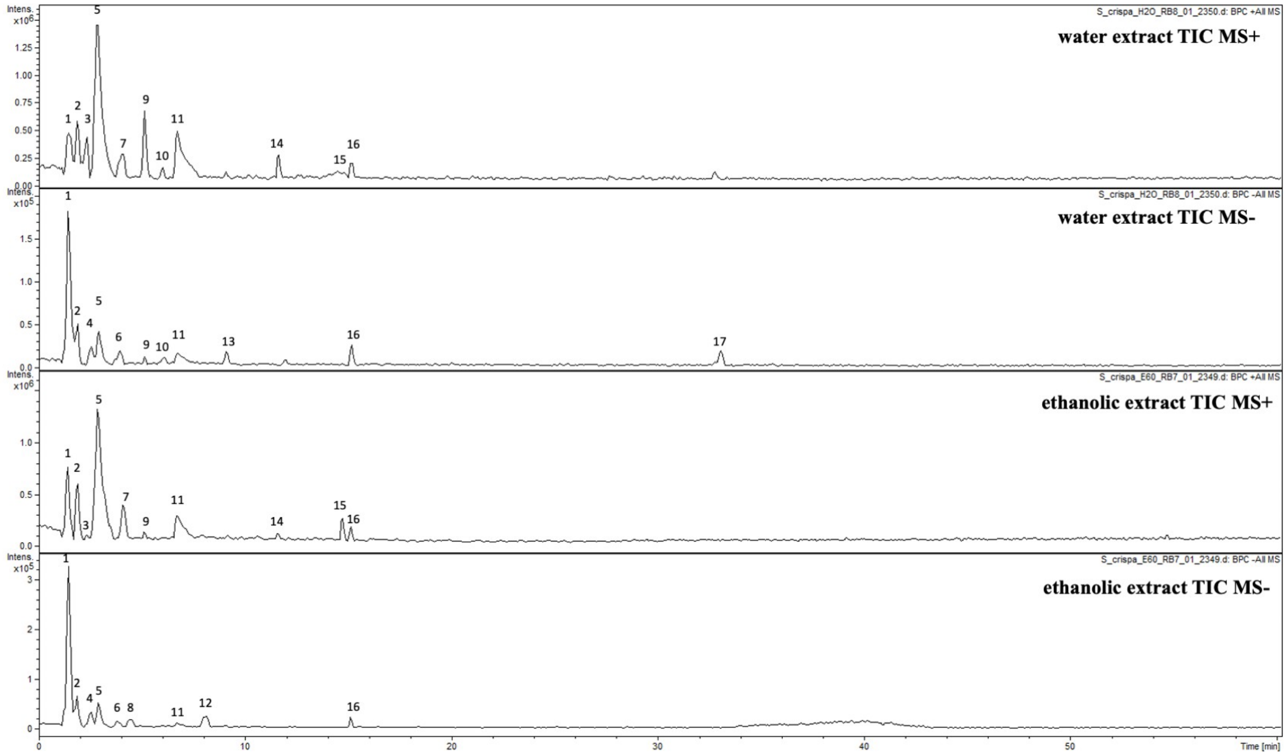

Figure S1. The UHPLC analysis of the chemical composition of water and 60% ethanolic extracts from *S. crispa.*
